# Which antipsychotics can we use for obsessive-compulsive symptoms in schizophrenia?

**DOI:** 10.1192/j.eurpsy.2021.1710

**Published:** 2021-08-13

**Authors:** A. Aissa, H. Ghabi, D. Khattech, S. Meddouri, U. Ouali, F. Nacef

**Affiliations:** 1 Psychiatry A, Razi Hospital, Manouba, Tunisia; 2 Psychiatry A Department, Razi Hospital, Manouba, Tunisia

**Keywords:** comorbidity, atypical antipsychotics, Obsessive-compulsive symptoms, schizophrénia

## Abstract

**Introduction:**

Obsessive-compulsive symptoms (OCS) are common in schizophrenia, with a prevalence ranging from 12 to 25%. They affect negatively disease outcome. Patients with comorbid OCS present more frequently resistant psychotic symptoms. Besides, the appearance and aggravation of OSC are more commonly reported with atypical antipsychotics.

**Objectives:**

To present through a clinical case and a brief literature review the treatment challenge of obsessive-compulsive symptoms in schizophrenia.

**Methods:**

We reported the case of Mr. M.S., treated in our department since 2008 for comorbid schizophrenia and OCS, and discussed therapeutic alternatives through a literature review.

**Results:**

Mr. M.S. a 34-year-old male diagnosed with comorbid schizophrenia and OCS at age 20. To control psychotic symptoms, the patient received several trials of anti-psychotics with little improvement. We concluded that it was resistant schizophrenia. The introduction of clozapine reaching 300 mg daily led to significant improvement of psychotic symptoms but worsened OCS. The adjunction of fluoxetine and cognitive-behavioral therapy (CBT) was unsuccessful to manage obsessive symptoms. We opted for the association of aripiprazole 20 mg daily and clozapine, the doses of which were gradually tapered down to 150 mg daily. This association has guaranteed the improvement of both psychotic and obsessive symptoms.

**Conclusions:**

Conclusion This clinical vignette highlights the need for clinical awareness about the possible exacerbation of OCS with atypical antipsychotics in schizophrenia.

**Disclosure:**

No significant relationships.

